# Toxicity of Organometal(loids)

**DOI:** 10.1155/2012/358484

**Published:** 2012-03-18

**Authors:** Elke Dopp, Shelley Bhattacharya, Alfred V. Hirner, Michael Aschner, Tanja Schwerdtle

**Affiliations:** ^1^University of Duisburg-Essen, Essen, Germany; ^2^Visva-Bharati University, Santinikrtan, India; ^3^Vanderbilt University Medical Center, Nashville, TN, USA; ^4^The University of Münster, 48149 Münster, Germany

Biomethylation of metals and metalloids is a process ubiquitously occurring in the environment (under aerobic and anaerobic conditions), which leads to the formation of chemical species with significantly higher mobility and altered toxicity. The alkylation of inorganic metal(loid)s through transfer, for example, of methyl groups, is a significant factor in the biogeochemical cycling of the metal(loid) elements. Biomethylation has been described in natural systems for arsenic, cadmium, germanium, mercury, sulfur, antimony, selenium, tin, tellurium, and lead as well as for bismuth, gold, chromium, palladium, platinum, and thallium under laboratory conditions.

In this special issue biomethylation products of arsenic, bismuth, mercury, lead, and tin are of special interest. Of all metal(loid) species in environmental systems, the element arsenic received the greatest attention worldwide. In this issue, recent research on influences of arsenic methylation on toxicity of arsenic species (M. Hall and M. Gamble), modes of action of arsenic metabolites in human cells (Bartel et al.), and the toxicity of volatile arsenic species compared to volatile species of bismuth, mercury, and tin (E. Dopp et al.) will be presented.

Anthropogenic water pollution by butyltin biocides is a well-documented and a severe environmental problem. Its distribution and accumulation in aquatic organisms and also within the food chain leads to biological effects in different organisms. The immunotoxic effects in mammalian cells is highlighted in this special issue by H. Krug.

Beside carcinogenic and immunotoxic effects, organometal(loid)s can exert neurotoxicity. The best known neurotoxic metal(loid) is methylmercury (MeHg). MeHg affects both, the developing and the mature central nervous systems. Several epidemics resulting from the consumption of food contaminated by MeHg have shown the disastrous effects on living organisms. Mechanisms associated with MeHg exposure and neurotoxic effects are described by P. Kaur et al. in this issue.

It has to be considered that humans not only are exposed to metal(loid) compounds from the environment via inhalation and ingestion, but may also be able to generate these species by endogenous enzymes or/and biomethylation in the colon. Methanoarchaea have an outstanding capability to methylate numerous metal(loid)s therefore producing toxic and highly mobile derivatives which might influence human health. Interesting studies in this field were carried out by the group of R. Hensel and new results are presented in this issue by B. Bialek et al. and B. Huber et al.

Metal(loid)-induced health effects, including carcinogenesis and neurodegeneration, have been reported in numerous publications. However, organisms and cells have developed protective mechanisms to deal with metal(loid) exposure. An overview about mechanisms involved in cellular detoxification of different metals is given in the review of E. Martinez-Finley and M. Aschner. Protein binding of metal(loid)s is also a possibility to detoxify serveral species, for example, lead. This process is highlighted in the review of H. Gonick.

Altogether, this special issue addresses contemporary concentrations of organometal(loids) increasing in dangerous proportions in our environment. Unknowingly, the human population is exposed to such insults, which on the long run may be a point of no return. It is true that there are mechanisms of detoxification which allow the biological systems to survive healthily. In spite of such innate mechanisms of combating stress, there is an urgent global need to realize the portent of environmental disaster staring at our face. Sooner we heed to the indications spelt out in this special issue better it is for the future of mankind.

*Elke Dopp* *Elke Dopp* *Shelley Bhattacharya* *Shelley Bhattacharya* *Alfred V. Hirner* *Alfred V. Hirner* *Michael Ashner* *Michael Ashner* *Tanja Schwerdtle* *Tanja Schwerdtle*

